# Obesity and Associated Lifestyle in a Large Sample of Multi-Morbid German Primary Care Attendees

**DOI:** 10.1371/journal.pone.0102587

**Published:** 2014-07-18

**Authors:** Claudia Sikorski, Melanie Luppa, Siegfried Weyerer, Hans-Helmut König, Wolfgang Maier, Gerhard Schön, Juliana J. Petersen, Jochen Gensichen, Angela Fuchs, Horst Bickel, Birgitt Wiese, Heike Hansen, Hendrik van den Bussche, Martin Scherer, Steffi G. Riedel-Heller

**Affiliations:** 1 Institute of Social Medicine, Occupational Health and Public Health, University of Leipzig, Leipzig, Germany; 2 Leipzig University Medical Center, IFB AdiposityDiseases, Leipzig, Germany; 3 Central Institute of Mental Health, Medical Faculty Mannheim/Heidelberg University, Mannheim, Germany; 4 Department of Medical Sociology, Social Medicine and Health Economics, Hamburg-Eppendorf University Medical Center, Hamburg, Germany; 5 Department of Psychiatry and Psychotherapy, University of Bonn, Bonn, Germany; 6 Department of Medical Biometry and Epidemiology, Hamburg-Eppendorf University Medical Center, Hamburg, Germany; 7 Institute of General Practice, Goethe-University Frankfurt am Main, Frankfurt am Main, Germany; 8 Department of General Practice, Jena University Hospital, Jena, Germany; 9 Department of General Practice, Medical Faculty, University of Dusseldorf, Dusseldorf, Germany; 10 Department of Psychiatry, Technical University of Munich, München, Germany; 11 Institute for Biometry, Hannover Medical School, Hannover, Germany; 12 Department of Primary Medical Care, Hamburg-Eppendorf University Medical Center, Hamburg, Germany; Bielefeld Evangelical Hospital, Germany

## Abstract

**Background:**

Obesity and the accompanying increased morbidity and mortality risk is highly prevalent among older adults. As obese elderly might benefit from intentional weight reduction, it is necessary to determine associated and potentially modifiable factors on senior obesity. This cross-sectional study focuses on multi-morbid patients which make up the majority in primary care. It reports on the prevalence of senior obesity and its associations with lifestyle behaviors.

**Methods:**

A total of 3,189 non-demented, multi-morbid participants aged 65–85 years were recruited in primary care within the German MultiCare-study. Physical activity, smoking, alcohol consumption and quantity and quality of nutritional intake were classified as relevant lifestyle factors. Body Mass Index (BMI, general obesity) and waist circumference (WC, abdominal obesity) were used as outcome measures and regression analyses were conducted.

**Results:**

About one third of all patients were classified as obese according to BMI. The prevalence of abdominal obesity was 73.5%. Adjusted for socio-demographic variables and objective and subjective disease burden, participants with low physical activity had a 1.6 kg/m^2^ higher BMI as well as a higher WC (4.9 cm, p<0.001). Current smoking and high alcohol consumption were associated with a lower BMI and WC. In multivariate logistic regression, using elevated WC and BMI as categorical outcomes, the same pattern in lifestyle factors was observed. Only for WC, not current but former smoking was associated with a higher probability for elevated WC. Dietary intake in quantity and quality was not associated with BMI or WC in either model.

**Conclusions:**

Further research is needed to clarify if the huge prevalence discrepancy between BMI and WC also reflects a difference in obesity-related morbidity and mortality. Yet, age-specific thresholds for the BMI are needed likewise. Encouraging and promoting physical activity in older adults might a starting point for weight reduction efforts.

## Introduction

Obesity is recognized as a major health threat throughout the life-span. It is highly prevalent in older individuals. Currently, about one third of all US adults above the age of 60 must be considered obese [1] and a further rise can be expected as those generations age that contributed to rising obesity rates during the last years [2]; [3].

Obesity in older adults is associated with an elevated risk for cardiometabolic syndromes, physical disability, impaired quality of life, and even dementia [4] as well as substantial functional impairment [5]. Recent studies showed an increase in absolute mortality risk up to the age of 75 [6]. The obesity paradox that has been described, namely a survival benefit of overweight and obese elderly, may mainly be a result of a positive survival bias and unintended weight loss that may be linked to life-threatening illness [4]. Many questions, however, remain unanswered and need further investigation. One aspect would be to investigate the association of senior obesity with other accompanying factors, such as lifestyle behavior. In certain unhealthy lifestyle choices were highly prevalent in obese elderly, a truly protective effect of senior obesity may be even more in question.

An obesogenic environment accompanied by changes in lifestyle factors, such as unfavorable nutritional intake and low physical activity, accounts for parts of the obesity pandemic [7]. The influence of these lifestyle factors on senior obesity in a population of over 65-year-olds has hardly been investigated. A study in a sample including participants aged 75 and older, showed age-related differences in fat mass to be associated with lifestyle factors. Low physical activity and unfavorable nutritional intake was associated with obesity in younger and in older respondents [8]. As obese elderly benefit from intentional weight reduction, it is necessary to determine influential factors on senior obesity [9]; [10].

This is one of the first studies analyzing the association with specific lifestyle behaviors with obesity in the elderly. Since general practitioners (GPs) are the major health care provider for elderly individuals and the primary care level turned out to be the setting to address lifestyle pattern in those most in need, this study is based on a large sample of multi-morbid primary care attendees. This comprises a most relevant group of individuals with more than 3 chronic conditions, which make up to three quarters of patients in primary care [11]. Research has shown that this kind of at-risk population benefits from behavioral interventions as well but studies on obesity and lifestyle in this population are lacking up to this date [12].

This study therefore sought to firstly determine the prevalence of overweight and obesity in a multi-morbid sample of German elderly primary care attendees aged 65 and above. The application of less age-dependent measures than BMI for the assessment of senior obesity has been discussed. As waist circumference (WC) as a surrogate of fat distribution (e.g. abdominal obesity) has been shown to be a more adequate predictor of impaired outcomes and elevated mortality in obese elderly [13], both, BMI and WC, will be considered as indicators of obesity. The association of individual lifestyle factors on BMI and WC is investigated.

## Methods

### Sample

The data were derived from the German MultiCare1 study investigating patterns of multi-morbidity in primary health care. At baseline, 3,189 multi-morbid subjects aged 65 to 85 years were included in the sample (mean 74.4 years, 59.3% women). For inclusion criteria, multi-morbidity was defined as being diagnosed with at least three out of a list of 29 diseases and syndromes. The study design and sample characteristics have been described in detail elsewhere [14]; [15].

### Data collection and assessment procedure

Between July 2008 and October 2009, participating GPs were interviewed regarding the patients' morbidity. All participating GPs received a thorough introduction to the study. Aside from the GP interview, GPs were asked to measure weight, height and waist circumference in each patient. Each questionnaire contained specific information where waist circumference was to be measured. WC was defined as the minimal circumference at the umbilicus in a standing patient.

Within the face-to-face patient interview with the participant, all relevant sociodemographic information was collected.

### Dependent variables

Body Mass Index was calculated by dividing the measured body weight by the squared body height in meters. It was then categorized according to guidelines (underweight or normal weight ≤24.9 kg/m^2^, overweight 24.9–29.9 kg/m^2^ and obese ≥30 kg/m^2^) [16]. Waist circumference was classified to be an indicator for abdominal obesity when it exceeded 102 cm for men and 88 cm for women [17]. These cut-points are associated with a significantly higher risk for metabolic and cardiovascular complications [18].

### Independent variables

Four different lifestyle behaviors and their association with obesity were investigated. They were assessed by a variety of instruments.

#### Physical activity

The International Physical Activities Questionnaire (IPAQ-S7S) was used to rate the participants' level of activity [19]. Reliability of the IPAQ across different study populations ranged from Spearman's Rho  = 0.66 to 0.88. For an elderly population the re-test reliability of this instrument was 0.65 and 0.57 for men and women aged 65 and older, but showed adequate validity [20]. All study participants were categorized as displaying low, moderate or high activity behavior. High activity behavior, for example, was defined in participants reporting vigorous-intensity activity on at least 3 days or a combination including vigorous-intensity activity on at least 7 days. Detailed information on the scoring procedure is provided by the IPAQ Research Committee [21].

#### Alcohol consumption

Alcohol consumption was determined by the AUDIT-C, a short screening test for alcohol disorders [22]. Alcohol consumption was classified according to gender-specific cut-off points. For men a score greater than 5 and for women a score greater than 2 was seen as high to risky alcohol consumption [23]. The AUDIT does not allow for a detailed analysis of alcohol units per week. One item asked the participants to state whether they never smoked, were former smokers or currently smoked.

#### Quality and quantity of food intake

Another lifestyle variable regarded food intake and nutritional behavior. A self-constructed scale consisting of 10 items was used to rate quantity of food (2 items: Meals per day and size of meal portions) and quality of food (8 items covering the different nutritional classes, such as dairy products, and their frequency of intake). Quantity of food was dichotomized – those that report to eat as proposed by the guidelines vs. those that eat too much (German Society for Nutrition) [24]. For each of the nine quality items, it was determined whether the amount of each nutritional class the participant consumed was guideline concordant (German Society for Nutrition) [24]. For example, participants were asked how many portions of dairy products they consumed during a day. The German Society for nutrition recommends three or more portions of dairy products. Respondents meeting that criterion scored one point on the self-constructed scale. A score ranging from 0 (not guideline concordant at all) to 8 (completely guideline concordant) was then dichotomized, including participants in the 75^th^ percentile in a guideline concordant, healthy eating group. The 75^th^ percentile started at a score of 4 points on the scale.

### Confounding variables

Age, gender and education served as socio-demographic confounding variables. Age was introduced as a continuous variable. Education was classified according to the CASMIN classification (low, moderate, high attainment) [25]. Additionally, subjective and objective disease burden were introduced as confounding variables since these may influence the impact of life-style factors. Objective disease burden was calculated as a score that included the number of co-morbid conditions weighted by the severity of these conditions. As obesity is the outcome variable in this study, obesity was excluded from the count of co-morbidities. Current subjective disease burden was ranked on the visual analog scale (VAS) within EuroQol (EQ-5D) Scale with a scale from 0–100 [26]. Functional status was assessed by the Barthel index. Impaired activities of daily living (ADL) were used to determine physical impairment of the participant [27]. The index consists of 10 items that are scored in three categories (0 points  =  high level of impairment, 5 points  =  medium level of impairment, 10 points  =  unimpaired). Respondents with a total of 100 points were categorized as completely unimpaired, 85 to 95 points as somewhat in need for care, 35 to 80 points in need for care, and below 30 points participants were rated as highly in need for care [27].

### Statistical analyses

In total, 3,127 participants (98%) had valid values for the BMI and were entered in cross-sectional analyses. Data on WC was available for 3,079 respondents (96%). In data cleansing, missing values were input for participants with extreme values (e.g. WC smaller than 50 or larger than 250 cm). Due to missing data on independent variables the number of complete cases for the full multivariate regression model amounts to 2,841 (89%).

All statistical analyses were performed using STATA 11.2 [28]. Chi-square test and oneway ANOVA and t-tests were used to test for significant proportion and mean differences, respectively. Different dependent variables (WC and BMI) were used as continuous variables in the linear regression models, adjusted for age, gender, education, objective and subjective disease burden as well as functional status. Margins were calculated from those models, representing the actual numerical difference in WC and BMI across different life-styles. Additionally, WC and BMI were used as categorical variables as described. For all analyses, “no response” codes were treated as missing values.

### Ethics approval

The study is conducted in compliance with the Helsinki Declaration. The study protocol was approved by the Ethics Committee of the Medical Association of Hamburg in February 2008 and amended in November 2008 (Approval-No. 2881). Written informed consent was given by all participants prior to the interview.

## Results

About one third of the participants (31.1%) were considered obese if BMI is taken into account. Women were more likely to be obese (34.0%) but less often overweight (38.3% compared to 51.1% in men). The mean BMI in men was 28.1 kg/m^2^ and 28.3 kg/m^2^ in women. Waist circumference categorization seemed more sensitive regarding abdominal obesity. Seventy-Three per cent of all participants had a waist circumference above the recommended level. An elevated WC was found in 79.4% of all women and 64.8% of all men. Men had a mean WC of 106.3 cm and women a mean WC of 98.4 cm. Almost all obese participants and even 62% of normal or underweight patients showed a waist circumference above the threshold (table 1).

**Table 1:** Baseline characteristics according to weight status.

	Non-obese	Obese	p
Men, % (n)	43.0 (927)	35.2 (342)	<0.001^a^
Age, years ± SD	74.6±5.3	73.9±5.0	<0.001^b^
Waist circumference above recommended threshold, % (n)	62.0 (1.312)	98.7 (944)	<0.001^a^
Educational attainment, % (n)			<0.001^a^
Low	59.9 (1289)	68.4 (665)	
Middle	27.9 (601)	23.9 (232)	
High	12.1 (261)	7.7 (75)	
Objective disease burden^c^, mean n ± SD	10.6±4.9	12.4±5.2	<0.001^b^
Subjective disease burden^d^, mean ± SD	64.2±17.8	58.5±18.4	<0.001^b^
Lifestyle factors			
Quality of food % (n)			0.678^a^
High (>75th percentile)	43.4 (914)	42.6 (403)	
Low (< 75th percentile)	56.6 (1194)	57.4 (544)	
Quantity of food, guideline concordant, % (n)	83.6 (1795)	84.2 (815)	0.660^a^
Physical activity, % (n)			<0.001^a^
Low	27.7 (585)	44.3 (427)	
Moderate	45.0 (951)	36.6 (353)	
High	27.3 (576)	19.1 (184)	
Smoking status, % (n)			<0.001^a^
Never	46.5 (1001)	51.9 (504)	
Former	42.6 (916)	42.8 (416)	
Current	10.9 (234)	5.4 (52)	
Alcohol consumption, % (n)			<0.001^a^
Abstinent	22.2 (477)	30.7 (297)	
Low to Moderate	39.3 (844)	38.2 (370)	
High	38.5 (827)	31.3 (301)	

Weight status as determined by Body Mass Index (BMI ≥30);

a comparison between obese and non-obese based on chi-square test;

b comparison between obese and non-obese based t-test,

c number of diseases weighted by severity;

d visual analogue scale.

Table 1 summarizes baseline characteristics of participants by general obesity status. Age, gender, educational attainment, mean lifestyle score, physical activity, smoking status, alcohol consumption and objective as well as subjective health perception were significantly associated with general obesity.

Baseline means of BMI and WC are reported in Table 2. In the analyses adjusted for potential confounders (e.g. age, gender and disease burden, model 2), subjects with a low level of physical activity had a higher BMI (+1.6 kg/m^2^, p<0.001) and WC (+4.9 cm, p<0.001) compared to those with a high level of activity. Participants with a high to risky alcohol consumption as well as current smokers showed lower BMI and WC compared to those that are abstinent from alcohol and tobacco. Eating behavior related variables were associated with neither BMI nor WC in the adjusted analyses.

**Table 2:** Baseline Means of BMI and WC according to lifestyle factors.

	Bivariate Analyses		Model 1^a^		Model 2^b^	
	Mean (95% CIs)	p	Mean (95% CIs)	p	Mean (95% CIs)	p
BMI (kg/m^2^)						
Quality of food						
High (>75^th^ percentile)	28.1 [27.9 to 28.4]	0.413	28.2 [27.9 to 28.4]	0.621	28.2 [28.0 to 28.4]	0.933
Low (<75^th^ percentile)	28.3 [28.1 to 28.5]		28.3 [28.0 to 28.5]		28.2 [27.8 to 28.5]	
Quantity of food						
Guideline concordant	28.2 [28.0 to 28.4]	0.742	28.1 [27.7 to 28.6]	0.703	28.2 [28.1 to 28.4]	0.564
Above guideline rec	28.2 [28.0 to 28.4].		28.2 [28.0 to 28.4]		28.1 [27.7 to 28.5]	
Physical activity						
Low	29.5 [29.2 to 29.8]	<0.001	29.6 [29.3 to 29.9]	<0.001	29.2 [28.9 to 29.5]	<0.001
Moderate	27.7 [27.5 to 28.0]	0.275	27.7 [27.5 to 28.0]	0.075	27.8 [27.6 to 28.1]	0.309
High	27.5 [27.1 to 27.8]		27.3 [27.0 to 27.7]		27.6 [27.3 to 28.0]	
Smoking status						
Never	28.4 [28.2 to 28.6]		28.4 [28.1 to 28.6]		28.4 [28.1 to 28.6]	
Former	28.4 [28.1 to 28.6]	0.883	28.4 [28.2 to 28.7]	0.902	28.4 [28.2 to 28.7]	0.801
Current	26.6 [26.0 to 27.1]	<0.001	26.4 [25.8 to 27.0]	<0.001	26.2 [25.7 to 26.8]	<0.001
Alcohol consumption						
Abstinent	28.9 [28.5 to 29.2]		28.8 [28.4 to 29.1]		28.5 [28.2 to 28.9]	
Low to Moderate	28.3 [28.0 to 28.6]	0.010	28.4 [28.2 to 28.7]	0.133	28.4 [28.1 to 28.7]	0.546
High	27.7 [27.4 to 28.0]	<0.001	27.6 [27.3 to 27.9]	<0.001	27.8 [27.6 to 28.1]	0.003
WC (cm)						
Quality of food						
High (>75^th^ percentile)	100.8 [100.1 to 101.5]	0.006	101.2 [100.5 to 101.9]	0.201	101.7 [101.0 to 102.4]	0.858
Low (<75^th^ percentile)	102.2 [101.5 to 102.8]		101.8 [101.2 to 102.4]		101.6 [101.0 to 102.2]	
Quantity of food						
Guideline concordant	103.2 [102.1 to 104.4]	0.002	101.7 [100.6 to 102.9]	0.757	101.6 [101.1 to 102.1]	0.891
Above guideline rec	101.3 [1000.7 to 101.8]		101.5 [101.0 to 102.0]		101.7 [100.6 to 102.8]	
Physical activity						
Low	105.1 [104.3 to 105.9]	<0.001	105.8 [105.0 to 106.6]	<0.001	104.7 [103.9 to 105.5]	<0.001
Moderate	100.0 [99.3 to 100.7]	0.652	100.0 [99.3 to 100.6]	0.069	100.4 [99.7 to 101.0]	0.322
High	99.8 [98.8 to 100.7]		98.9 [98.0 to 99.8]		99.8 [98.9 to 100.7]	
Smoking status						
Never	99.9 [99.2 to 100.5]		101.4 [100.7 to 102.0]		101.5 [100.8 to 102.2]	
Former	103.8 [103.1 to 104.5]	<0.001	102.4 [101.7 to 103.1]	0.054	102.4 [101.7 to 103.1]	0.097
Current	100.5 [98.9 to 102.0]	0.459	99.4 [97.9 to 100.9]	0.018	98.9 [97.4 to 100.4]	0.002
Alcohol consumption						
Abstinent	102.4 [101.4 to 103.3]		103.2 [102.3 to 104.1]		102.5 [101.6 to 103.4]	
Low to Moderate	103.6 [102.9 to 104.4]	0.041	101.8 [101.1 to 102.6]	0.027	101.8 [101.0 to 102.5]	0.239
High	98.8 [98.0 to 99.6]	<0.001	100.2 [99.4 to 101.0]	<0.001	100.9 [100.1 to 101.7]	0.008

Bivariate analyses and adjusted analyses for each lifestyle factor; BMI – Body Mass Index; WC – Waist circumference;

a adjusted for age, sex, educational attainment;

b adjusted for age, sex, educational attainment, subjective and objective disease burden and need for care.

In table 3 the full logistic models showing effects of differentiated health behaviors on categorical WC and BMI are displayed. Current smokers had lower probability of elevated BMI (OR  = 0.377, p<0.001) but not WC (OR  = 0.740, p = 0.060). Former smokers, however, displayed a higher WC (OR  = 1.311, p = 0.009). High to risky alcohol consumption was associated with lower probabilities of obesity for both outcomes. Again, low physical activity was associated with a lower probability of elevated BMI and WC. Food quantity and quality remained without association. The models with individual health behaviors as independent variables accounted for 7.1% (BMI) and 6.0% (WC) of variance.

**Table 3:** Logistic regression BMI and WC with individual lifestyle behaviors^a^.

	Model 1	Model 2
Independent variables	BMI obesity Odds Ratio	WC obesity Odds Ratio
Level of activity (ref = high)		
Moderate activity	1.151	1.310
Low activity	1.988***	1.906***
Drinking behavior (ref = abstinent)		
Low to moderate consumption	0.894	0.860
High to risky consumption	0.699**	0.765**
Smoking status (ref = never)		
Former smoker	0.988	1.311**
Current smoker	0.377***	0.740
Quantity of food (ref = too little or guideline concordant)	0.973	0.988
Quality of food (score)	1.007	0.991
Constant	1.156	2.920***
Observations	2841	2802
Adjusted *R* ^2^	0.071	0.060

**p<0.01,

***p<0.001.

a adjusted for age, sex, educational attainment;

Abbreviations: BMI – Body Mass Index; WC – Waist circumference.

## Discussion

This cross-sectional study set out to determine the prevalence of overweight and obesity assessed by different anthropometric measures in a multi-morbid sample of German elderly (65+) and to investigate factors associated with general and abdominal obesity with a special focus on lifestyle related behaviors. It finds differences in the prevalence of obesity according to BMI or waist circumference. Physical activity is clearly associated with a lower BMI and WC, even when controlling for disease burden and socio-demographic variables.

Three out of four participants within this study were affected by either overweight or obesity. A recent study was able to show similar prevalence rates. Our findings are almost an exact replication of the prevalence found in the elderly German population (National Nutrition Survey II, Nationale Verzehrsstudie II; 31% total prevalence of obesity, 43.5% overweight), although data was obtained in a multi-morbid sample [29]. The distinctiveness of multi-morbidity might be reflected in the high numbers of elevated waist circumference in our sample. In the general population only 44.5% (men, 60- to 69-years old) and 57% (70+) had a WC above the recommended threshold. In our sample that number was exceeded substantially (65%). About 50 to 60% of women in the general public had an elevated WC, compared to 80% in this sample. This is of special importance as it is known that women are prone to accumulate abdominal fat after menopause that may affect adverse outcomes, such as the incidence of diabetes [30]–[32].

WC might capture elevated risks of cardiovascular and metabolic conditions, which are known to be highly prevalent in multi-morbid samples [33], more adequately than BMI. Since the WC cut-offs, but not the BMI cut-offs, were set specifically at the point of elevated risk for these conditions, the higher prevalence of increased WC may not be surprising. These results go in line with a previous study, where the authors reported a prevalence of 47% for general obesity and 73% for abdominal obesity in a sample of multi-morbid participants [34]. This assumption is supported by a study of Spanish elderly of the general population (not specifically multi-morbid). There, prevalence numbers of central obesity are about the same as in our study, while abdominal obesity prevalence is higher (totaling 56%) but not to the extent as it was seen in the multi-morbid samples [35].

Alternatively, a measurement error in WC can be an explanation for the large deviance we find. WC is particular is not the easiest and most reliable measure of obesity, especially in the elderly. A recent study found that the point of measure (narrowest point between the inferior rib border and the iliac crest) for WC may be difficult to find in the obese elderly which made up quite a proportion of this sample [36]. The measurement error can range from 0.7 cm to 15 cm [37] and this study lacks data for quality checks. Although WC measurement can therefore not be considered to detect small changes following interventions, its use can be valuable in epidemiological studies as this [38]. Furthermore, the lack of missing values as well as extreme outliers can be a potential indicator for valid data in this study.

Individual lifestyle choices have been shown to be significantly associated with the existence of obesity (e.g. [39]) and changing lifestyle patterns have shown to have effects on e.g. cognitive health [40]. This study therefore emphasizes the relevance of modifiable lifestyle factors in the development and maintenance of overweight and obesity. Although causal conclusions cannot be drawn from the cross-sectional study, controlling the effect of life-style factors for socio-demographic and disease related effects, eliminates some alternative explanations.

High to moderate physical activity was inversely associated with BMI and WC values. Obviously, a higher energy expenditure via physical activity balances out the energy homeostasis. This is of special importance since the resting metabolic rate is said to decrease by 2–3% every life year [41], thus making physical activity crucial in maintaining a constant weight. A study was able to show that a diet accompanied by physical activity was most successful in weight loss efforts in a sample of 65-year-olds [42]. A review in adolescents suggests physical activity to act as a protective factor in obesity [43], but existent obesity might be a barrier to physical activity as well. A decrease in energy expenditure rather than an increase in energy intake is described to be responsible for the increase of total fat mass with age [41]. This decrease in energy expenditure is partly due to a decreased level of physical activity, suggesting that efforts to keep physical activity high even in older age might influence weight and fat mass [42]. Recent studies even show a beneficial effect of commenced physical activity in old age on the incidence of dementia, underlying the importance of physical activity [44]; [45].

It is difficult to understand the observed association between current smoking and lower BMI values. The present findings are in agreement with a previous report about Spanish elderly [34], but in contrast to what has been reported in Switzerland [46]. In current smokers, nicotine increases energy expenditure, but heavy smoking might also be associated with other obesogenic behaviors, suggesting an u-shaped association [46]. A prospective study showed that active smokers and quitting smokers had greater weight gains over a follow-up period of 50 months compared to those who did not smoke [47]. This fining in particular emphasizes the importance of attempt to reduce smoking at a population level as it seems to be a risk factor even for obesity. Current smoking may, via increased energy expenditure, positively influence weight status, but poses, in the long run, a risk factor.

The decrease in body size with increase of alcohol intake has been shown before, however, explanations are lacking [48]. This seems of special importance since this study used gender-specific cut-off values to determine high to risky consumption levels according to guidelines [23]; [49]. We were not able, however, to determine the kinds of drinks consumed which is one important factor discussed when evaluating the influence of alcohol consumption [48].

Dietary variables did not show significant effects on BMI or WC. Using a combined score to determine guideline concordant eating behavior may have resulted in a loss of relevant information, however, in post-hoc analyses, the individual influence of nutritional components (such as fruit and vegetable intake) was assessed, but did not yield clear associations either. For example, eating more portions of fruit every day, was associated with a lower likelihood of being abdominal obese (OR  = 0.93, p = 0.040) but not general obese (OR  = 0.96, p = 0.263). The effects vanish when controlling for educational attainment. This seems to support the assumption that individual eating choices are not as much of relevance as eating patterns, such as the Mediterranean Diet that has been shown to be associated with lower obesity prevalence [34]. Standardized scales to assess nutritional intake ought to be used. In depth investigation of the role of quality and quantity of food with objective assessment is obviously still needed.

### Strengths and Limitations

This study has several strengths and limitations. It provides the first basis of data on obesity in multi-morbid senior citizens in German primary care. The large sample of participants increases likelihood of reliable results. Although the majority of GP patients in Germany can be considered multi-morbid, the selection of patients may have lead to a bias of the association between physical measures and health outcomes.

One important aspect that needs to be considered is the potentially limited validity of waist circumference measure in general practices. Although a specific instruction was in place and all GPs were thoroughly instructed, measurement errors cannot be ruled out. The kind of instruction that was used was easy to understand and was easily implemented. Previous research shows that the kind of protocol that is used has no influence on the prospective associations with mortality and morbidity [50].

The lifestyle factors that were included can be assessed easily which might be of importance when translating findings into prevention or intervention efforts. Obviously, a more in depth assessment of lifestyle factors might have contributed to a more detailed understanding of mechanisms. Furthermore, the self-report of e.g. food intake might be less reliable than an experimental assessment. There is no data available on the validity of the food scale that was used; however, it is closely related to previous food frequency questionnaires (FFQ). While the validity of these questionnaires declines with the length of the food list, this relatively short instrument may have an advantage. Coherence and grouping of the items fulfill criteria of valid FFQs [51]. Also, the IPAQ questionnaire has not been fully evaluated in elderly samples yet. Its moderate reliability will need further investigation; however, correlation with objective measures (accelerometer) was satisfactory in previous studies [20]. Also, retrospective data on weight course and nutrition especially during adulthood was not assessed. Because of the cross-sectional design of these analyses, causal relationships can only be hypothesized but not proven; however, longitudinal data from the same study will be available to enlighten open questions. These cross-sectional analyses provide first information and potentially associated variables that can now be investigated in follow-up studies.

Furthermore, the explained variance of the regression models was limited. Considering the complexity of BMI and WC determinants, however, we feel that it was sufficient. Obviously, including information on past weight, as well as genetic markers in the analyses would have further increased the level of explained variance.

### Conclusions

Especially in multi-morbid patients, the prevalence of elevated waist circumference and obesity according to BMI differs substantially. Waist circumference might be an even more sensitive marker for obesity than the BMI. Age-specific thresholds for the BMI are needed which can only be assessed through prospective studies. Of all lifestyle factors that were investigated, physical activity was the only one with a clear association to lower BMI and WC values. Motivating older adults to stay active seems crucial. In multi-morbid patients, one approach to achieve that goal may be through their general practitioner.
